# Vestigial-like 4 Regulates Neurogenesis and Neural Crest Formation During *Xenopus* Development

**DOI:** 10.3390/jdb14010008

**Published:** 2026-02-11

**Authors:** Pierre Thiébaud, Emilie Simon, François Moisan, Sandrine Fedou, Hamid-Reza Rezvani, Nadine Thézé

**Affiliations:** Univ. Bordeaux, INSERM, BRIC, U 1312, F-33000 Bordeaux, France; pierrethiebaud1@gmail.com (P.T.); emsimon56@yahoo.fr (E.S.); francois.moisan@u-bordeaux.fr (F.M.); sandrine.fedou@u-bordeaux.fr (S.F.); hamid-reza.rezvani@u-bordeaux.fr (H.-R.R.)

**Keywords:** vestigial-like, neurogenesis, neural crest, TEAD, YAP, *Xenopus*

## Abstract

VESTIGIAL-LIKE proteins constitute a family of evolutionarily conserved proteins that act as cofactors in regulating gene expression through their binding to TEAD transcription factors. Among the four members of this family in vertebrates, VESTIGIAL-LIKE 4 has emerged as a tumor suppressor that competes with YAP in binding TEADs, thus inhibiting the HIPPO pathway downstream of YAP. Nevertheless, very few studies have addressed its function during early vertebrate development. Here, we used gain- and loss-of-function strategies to investigate the role of vestigial-like 4 during *Xenopus laevis* development. Our data show that vestigial-like 4 is a key regulator of neurogenesis and neural crest formation. In embryos depleted of vestigial-like 4, neurogenesis is severely impaired, and neither neurog1 nor neurod1 is able to stimulate neurogenesis. Vestigial-like 4 is also required for neural crest formation through *pax3* and sox9 regulation, and this property does not necessarily require its interaction with tead. Collectively, our findings demonstrate that vestigial-like 4 is an important regulator of neurogenesis and neural crest formation. Although vestigial-like 4 can bind to tead proteins in the embryo, its function does not depend solely on this interaction, suggesting a complex level of regulation with which vestigial-like 4 regulates early steps in development and differentiation.

## 1. Introduction

The VESTIGIAL LIKE (VGLL) family of proteins originated from the founding member vestigial (Vg), which was first characterized in *Drosophila melanogaster*. In *Drosophila*, the *vestigial* gene (*vg*) is required for the formation of wings and can induce wing outgrowth on other structures [1,2]. Vestigial proteins are nuclear proteins that lack a nucleic acid domain but form a transcriptional activator complex with the protein scalloped (Sd), a member of the TEAD family of transcription factors. Vestigial proteins are categorized as selector proteins that promote wing development and the regulation of wing-specific gene expression [1,2,3,4,5,6]. The first identified vertebrate homologue of vestigial proteins was the mammalian protein TONDU [7]. TONDU has been renamed VESTIGIAL LIKE 1 (or VGLL1) and is a 258 amino acid protein whose homology with the *Drosophila* protein spans a 24 amino acid region, or the Tondu domain (TDU), that mediates the interaction with TEAD proteins. Since then, several *VGLL* genes have been identified in vertebrates, all of which share a TDU domain [8,9,10,11,12]. This domain is very ancient and has a premetazoan origin [13]. Currently, VGLL proteins fall into two subfamilies. The first one corresponds to the vertebrate proteins VGLL1, VGLL2 or VGLL3, which, like *Drosophila* Vestigial, have a single TDU domain. The second subfamily corresponds to proteins that contain two TDU domains, such as VGLL4 in vertebrates or its *Drosophila* homologue Tgi [13,14,15]. An additional VGLL4 protein, named Vgll4L, has been described in zebrafish and *Xenopus* [16]. The protein has only 30–40% identity with *Xenopus* Vgll4, except over the TDU domains, which are completely conserved. The absence of *Vgll4L* genes in chickens, mice or humans suggests that they are homeologs that have evolved from a duplication event in the shared lineage to fishes and amphibians. The Tondu domain is the signature of VGLL proteins and is essential for their interaction with the TEAD family of transcription factors [7,17]. Outside the TDU domain, the proteins show little or no homology. In the VGLL1-3/Vg subfamily, the unique TDU domain is 24 amino acids long, whereas in the VGLL4/Tgi subfamily, the two TDU domains (TDU1 and TDU2) are 10 amino acids long and are highly conserved from sponges to mammals. Because TDU1 and TDU2 are found early in evolution before the appearance of TDU in bilaterians, they may be considered the ancestral TDU domain and constitute the basic evolutionary unit of VGLLl proteins [15].

Although many studies have focused on the function of Vestigial in *Drosophila* since its description, the roles played by vertebrate orthologues in development have been little explored to date. In addition to its role in wing formation, Vg has also been shown to regulate muscle development and differentiation in *Drosophila*, and this property requires its interaction with the key myogenic factor Dmef2 [18,19,20]. This property seems to be evolutionarily conserved, as mammalian VGLL2 has been found to be an essential cofactor of TEAD that is able to stimulate muscle differentiation induced by MyoD or interact with MEF2 to activate MEF2-dependent promoters [11,21]. VGLL2 function has also been investigated in zebrafish embryos, and morpholino-based knockdown analysis revealed that VGLL2 is required for the survival of the pharyngeal endoderm and plays a role in the development of the neural crest cell-derived craniofacial skeleton [22].

Human VGLL4, the first protein identified with two Tondu domains, has been shown to regulate α-1 adrenergic-dependent gene expression [9]. In these experiments, VGLL4 was found to be a negative regulator of both α-1 adrenergic and skeletal actin promoter transcription mediated by TEAD1 [9]. More recently, findings from several studies have shed new light on the function of VGLL4. In *Drosophila*, Tgi the homologue of human VGLL4, has been characterized in screens seeking genes that regulate the Hippo pathway or aiming to identify new Sd partners [13,14]. Tgi stands for the Tondu domain-containing growth inhibitor, and accordingly, it was demonstrated to act as a repressor of the Hippo pathway by directly binding to Sd, thus inhibiting the transcriptional activation of the Sd-Yki complex [13,14]. Human VGLL4 can phenocopy Tgi-dependent suppression of tissue overgrowth when overexpressed in *Drosophila* and antagonize the Hippo pathway in a transgenic mouse model of hepatocellular carcinoma induced by YAP (mammalian Yorkie orthologue) overexpression [13]. Since these findings, several reports have confirmed the antioncogenic functions of VGLL4 in various models, a property that is also shared by other VGLL members [23,24,25,26,27,28,29]. Moreover, Vgll4 has been shown to play a role in muscle regeneration and heart valve development in knockout mice [30,31]. Despite these works, very little is known about the functions of VGLL4 during embryonic development. In zebrafish embryos, Vgll4 has been found to be essential for normal epiboly during gastrulation [32]. Moreover, Vgll4 is sensitive to the oxygen concentration and regulates erythropoiesis differentiation [33]. With respect to the *Xenopus* model, we characterized only two *tead* genes and four *vgll4* genes [10,34]. Among the amphibian *vgll* gene family, *vgll4* is the only gene that is maternally expressed. *Vgll4* and *vgll4L* are expressed mainly in the neural plate and neural folds [10], whereas vgll4L is expressed predominantly in the neural folds and epidermis [16]. Later, the expression of both genes was strongly enhanced in neural tissue, anterior neural plate, migrating neural crest, and optic vesicles [10,16].

Here, we used gain- and loss-of-function strategies to address the role of vgll4 during neurogenesis and neural crest formation in *Xenopus* embryos. We show that morpholino knockdown of *vgll4* impairs early neural development. We found that neuronal differentiation induced by neurogenin 1 overexpression is dependent on vgll4. We provide evidence that vgll4 regulates the expression of *pax3*, a major regulator of neural crest development. Although we found that vgll4 can interact with tead proteins in the embryo, mutational analysis showed that vgll4 functions are not strictly dependent on its TDU domains. Taken together, our results demonstrate that vgll4 regulates different pathways during early development, providing important insights into factors that play key roles in neural development and neural crest formation.

## 2. Materials and Methods

### 2.1. Plasmid Constructions

*Vgll4.La* was generated via polymerase chain reaction (PCR) and cloned and inserted into the pCS2HA vector. *Vgll4.Lb* was obtained via 5′RACE via the Invitrogen^TM^ system (Thermo Fisher Scientific, Carlsbad, CA, USA) and was subsequently cloned and inserted into *pCS2-HA*. *Vgll4.Sa* was obtained from embryos via RT–PCR and subsequently cloned and inserted into *pCS2-HA*. All the vgll4-deleted forms were obtained via PCR and subsequently cloned and inserted into *pCS2-HA*. *Vgll4.La-mutTDU* (V4mutTDU) construct, in which both the TDU1 and TDU2 domains have been mutated, was obtained via Q5 site-directed mutagenesis (Biolabs, Cambridge, MA, USA), which enables site-specific mutagenesis in double-stranded plasmid DNA according to the manufacturer’s instructions. The core domain of TDU 1/2 is completely conserved between the human and *Xenopus* vgll4 proteins [15], and according to Koontz et al. [13] and Guo et al. [14], TDU1 and TDU2 mutations are made by changing the conserved EEHF sequence at the core of the domain to AAAA. This mutation completely abolishes the binding of VGLL4 to TEAD proteins [13,14]. The *Tead1*-myc constructs were obtained by subcloning the original clones (see [34]) into the *pCS2-Myc* vector. *Vgll4-VP16* and *vgll4-EnR* were generated by subcloning *vgll4*. The *vgll4.La* coding sequence was inserted into the *pCS2-VP16* and *pCS2-EnR* vectors after PCR amplification. For the in vivo assay of morpholino efficiency, a ~100-nucleotide 5′ fragment of vgll4 was used. *vgll4.La* or *vgll4.Sa* were inserted in frame into pCS2-GFP to produce the corresponding capped mRNAs. Details on the cloning procedure are available upon request. All the constructed plasmids were verified by sequencing.

### 2.2. Embryos, Injections, Treatments and Animal Cap Explant Culture

*Xenopus laevis* embryos were obtained as described previously [35]. The embryos were staged according to Nieuwkoop and Faber (NF) [36]. All protocols requiring *Xenopus* were carried out in accordance with the European Community Guide for Care and Use of Laboratory Animals and approved by the “Comité d’éthique en expérimentation de Bordeaux”, N° 33011005-A. In microinjection experiments, embryos were injected in one blastomere at the two-cell stage (NF stage 2) with mRNAs synthesized in vitro via the Message Machine Kit (Ambion, Austin, TX, USA). The amounts of mRNA injected were as follows: *Vgll4* (0.1–1 ng), *vgll4mutTDU* (1 ng), *vgll4Δ145 N* (1 ng), *vgll4Δ190C* (1 ng), *tead1* (c-myc tag, 50 pg) and *tead2* (c-myc tag, 50 pg), *Vgll4.La-GFP* or *vgll4.Sa-GFP* (50 or 200 pg). For the knockdown experiments, morpholino antisense oligonucleotides (MOs) were supplied by GeneTools Inc. (Philomath, OR, USA), as follows:

*vgll4.La* MO (V4LaMO): 5′-GGAGATGCGGCCCCGGTAACAAAAG-3′,
*Vgll4.La* mismatch MO (V4LaMM): 5′-GGACATCCGCCCCCGCTAAAAAG-3′,
*Vgll4.Lb* (V4LbMO): 5′-AACATCCAACGGATTCTCCATTCCT-3′,
*Vgll4.Sa* (V4SaMO): 5′-GCAGATCCATCTTCACACAGAGCAT-3′,
*tead1* MO (T1MO): 5′-CTCCAACTGCTCGGCTCCATGATTG-3′,
*tead2* MO (T2MO): 5′-AGGGTAAGGAGAGGCTCCTCCAAGC-3′,
*control* MO (cntrl MO): 5′-CCTCTTACCTCAGTTACAATTTATA-3′.

Preliminary experiments were performed to determine the effective dose of MOs that was set to 10–15 ng of vgll4 MO. For the rescue experiments, 1 ng of *vgll4.La* mRNA encoding a nontargeted mRNA was co-injected with the MOs. For all the injections, 250 pg of *β-galactosidase* mRNA was co-injected as a lineage tracer. For the animal cap explant experiments, both blastomeres of two-cell-stage embryos were injected into the animal pole region. The animal caps were then dissected at the blastula stage (NF stage 9) and cultured until the appropriate stages before RNA extraction. Animal cap assays, RNA extraction and RT–PCR analysis were performed as previously described [34] with the following oligonucleotides: Vgll4.La F, 5′-ATTGGTTCACGCAGATGACG-3′; R, 5′-ACGTAATCACAGAAGGTCG-3′; Vgll4.Lb F, 5′-GCTCAACTACCAGTATCTGC-3′; R, 5′-CACGTAATCACAGAAGGTCG-3′; Vgll4.Sa F, 5′-GAATCTCTCGGGAAGACTCG-3′; R, 5′-CACGTAATCACAGAAGGTCG-3′; Odc, F, 5′-GTCAATGATGGAGTGTATGGATC-3′; R, 5′-TCCATTCCGCTCTCCTGAGCAC-3′; all results shown are representative of three independent experiments.

### 2.3. In Situ Hybridization and Immunostaining

In situ hybridization was carried out as previously described [37] with full-length antisense probes labelled with digoxigenin corresponding to the indicated gene sequences. The chromogenic reaction was performed at room temperature in BM purple (Roche, 11442074001). The embryos were finally bleached in 10% hydrogen peroxide and 5% formamide in PBS before imaging. At the end of the experiments, all the data were analyzed independently by three people, and each of them scored the embryos under a stereomicroscope. All three examiners obtained the same score unambiguously. None of the embryos had an ambiguous phenotype that could confuse interpretation.

### 2.4. Protein Extraction, Western Blot, Immunoprecipitation and In Vitro Translation

Once the stage of development was reached, the embryos were lysed directly in cold RIPA buffer (PBS, 1% Triton, 1% NP40, 0.05% SDS, 1 µg/mL protease inhibitor cocktail (Roche, Basel, Switzerland), 1 mM Pefabloc, 20 mM NaF, and 1 mM Na vanadate) (20 µL per embryo with a minimum of 10 embryos). After mixing, the homogenate was sonicated 6 times for 15 s. To remove the yolk, two volumes of Freon (1,1,2-trichlorotrifluoroethane, Sigma, Merck, Darmstadt, Germany) (40 µL per embryo) were added, followed by vortexing. After 10 min of centrifugation at 13,000× *g* and 4 °C, the supernatant was recovered and 0.5 volume of 4× Laemmli sample buffer (Bio-Rad, Hercules, CA, USA) was added. The samples were then boiled for 5 min and stored at −80 °C. Proteins extracted from the equivalent of one embryo were separated via 12% SDS–PAGE and transferred to nitrocellulose membranes. Proteins were reacted with the following antibodies [mouse anti-HA (Covance, Princeton, NJ, USA, clone 16B12, 1/1000), mouse anti-c-myc tag (Sigma, 1/7000) and mouse anti-GFP (1/5000)] as recommended by the manufacturers. The blots were then probed with an HRP-conjugated goat anti-mouse secondary antibody and incubated to visualize the signal via an enhanced chemiluminescence detection kit (GE Healthcare, Fisher scientific, Illkirch, France).

For immunoprecipitation, embryos were injected in both blastomeres into the animal pole region at the two-cell stage (NF stage 2). Once the stage of development was reached, 20–30 injected embryos were frozen on dry ice prior to lysis in 50 mM Tris-HCl (pH 7.5), 150 mM NaCl, 0.5% Nonidet P-40 together with 1 mM PMSF and a proteinase inhibitor cocktail (Roche). Solubilized proteins were precleared with protein A Sepharose beads (Sigma) and incubated with appropriate antibodies (2 µg) [mouse anti-HA (Covance, clone 16B12) or mouse anti-c-myc tag (Sigma)] for 1 h at 4 °C and then incubated with protein A Sepharose beads for 1 h at 4 °C. The agarose beads were spun and washed in cold lysis buffer several times and then boiled in SDS sample buffer before being loaded onto 10% SDS–PAGE gels. Proteins were immunoblotted and stained with anti-c-myc (1/7000) or anti-HA (1/1000) antibodies. Bound antibodies were detected with HRP-conjugated secondary antibodies (EasyBlot kit anti-mouse IgG; Gene Tex, Irvine, CA, USA) diluted 1/1000 in EasyBloker solution (Gene Tex) to minimize background protein A contamination and visualized via an enhanced chemiluminescence detection kit (GE Healthcare, Chicago, IL, USA).

### 2.5. Alcian Blue Staining

Stage 47 larvae were fixed in MEMFA for 1 h at RT and dehydrated in 100% ethanol. Fixed embryos were incubated in acidic alcohol (80% ethanol, 20% acetic acid) containing 0.05% Alcian blue (Sigma) for 3 days and then washed in 95% ethanol for 15 min. The embryos were rehydrated with 75% to 25% ethanol mixed with 2% potassium hydroxide (KOH) and washed in 2% KOH. The embryos were transferred to 2% KOH containing a progressive increase in glycerol (20–60%) for 1 h each and finally washed in 80% glycerol in 2% KOH overnight at RT. The embryos were dissected to visualize the cartilage structure in the head.

### 2.6. TUNEL and pHH3 Staining

TUNEL staining was performed according to a previously described protocol [38]. Briefly, embryos were fixed in MEMFA (100 mM MOPS, pH 7.4; 2 mM EGTA; 1 mM MgSO_4_; and 4% formaldehyde) and stored in methanol at −20 °C. The embryos were rehydrated in PBT (0.2% Tween 20 in PBS), washed in PBS, and incubated with 150 U/mL terminal deoxynucleotidyl transferase (Roche) and 0.5 µM digoxigenin-dUTP (Roche). The reaction was terminated in PBS/1 mM EDTA at 65 °C, followed by washing in PBS. Detection and chromogenic reactions were carried out according to Harland [38]. The embryos were blocked in PBT + 20% goat serum, followed by incubation with an anti-digoxigenin antibody coupled with alkaline phosphatase (Roche). The embryos were washed in PBS and stained with nitro blue tetrazolium and 5-bromo-4-chloro-3-indolyl phosphate substrates. The reaction was visible within 30 min, and the embryos were viewed following dehydration in methanol. For phosphohistone H3 (pHH3) detection [39], embryos were incubated successively with an anti-pHH3 antibody (Upstate Biotechnology, 06-570; 1 μg/mL, Merck, Darmstadt, Germany) and anti-rabbit IgG secondary to alkaline phosphatase (Thermo Fisher Scientific, G-21079; 1:1000, Waltham, MA, USA). pHH3-positive cells were quantified according to a previously published method [40].

### 2.7. Luciferase Activity Measurement

Embryos were injected with *tead1*, *yap* and *vgll4* mRNAs together with the plasmid pGL284LUC, which contains 284 bp of the 5′ upstream region of the *Xenopus* tropomyosin gene cloned in the luciferase reporter gene pGL3-basic [41]. The plasmid pRL-TK was used for normalization (Promega, Madison, WI, USA). The animal caps were dissected from stage 9 embryos and cultured for 3 h before being frozen in liquid nitrogen. Luciferase activity was determined from 10 µL of the supernatant, according to the manufacturer’s instructions (Dual Luciferase Kit, Promega) and quantified with a Varioskan^®^ Flash (Thermo Fisher Scientific, Waltham, MA, USA). Relative firefly luciferase activity (RLU) was normalized to Renilla luciferase activity, and the results were calculated from duplicate samples of three independent repeats. The quantitative data are presented as the means ± s.e.m.s and were analyzed via Student’s unpaired two-tailed test. Statistical significance was defined as *p* < 0.05.

## 3. Results

### 3.1. The Xenopus Vgll4 Gene Is Structurally Equivalent to Its Mammalian Orthologues and Produces Two Transcripts That Are Differentially Regulated During Development

In a previous work, we reported the identification of four members of the *vgll* gene family in *Xenopus* and their expression during development [10,16]. *Vgll4* is the only member of the family that is maternally expressed with a constant expression level throughout development [10,16]. We initially characterized a *vgll4* mRNA (*vgll4.La*), which encodes a 293 amino acid protein. A second *Xenopus laevis vgll4* mRNA (*vgll4.Sa*) is present in databases and has 93% identity with *vgll4.La*. The two distinct mRNAs correspond to the products of *vgll4* homeolog genes, *vgll4.L* and *vgll4.S*, as expected from the allotetraploid nature of the *Xenopus laevis* genome and its two subgenomes [42]. Synteny analysis confirmed that *vgll4.La* is a homologue of mammalian *VGLL4* genes, whereas *vgll4.Sa* corresponds to the duplicated gene (Figure S1A). According to databases, the mammalian VGLL4 gene possesses two alternative promoters allowing the production of two proteins with distinct N-terminal ends [15]. We wondered whether such an isoform was also produced by the amphibian *vgll4* genes. We cloned a cDNA whose sequence was completely identical to the cDNA sequence *vgll4.La* via a rapid amplification of cDNA ends (RACE) but that diverge in its 5′ sequence. The corresponding mRNA encodes a 286 amino acid protein, which we named vgll4.Lb, with a unique 22 amino acid N-terminal end (Figure S1B). These findings suggest that two distinct mRNAs can be produced by the amphibian gene. To clarify this situation, we retrieved *vgll4* genomic sequences from a data bank and their comparison with cDNA sequences allowed us to establish the complete genomic organization of the *Xenopus vgll4* gene. The *Xenopus vgll4* gene is structurally identical to its mammalian orthologues with 6 exons and two promoters and an identical intron/exon splicing pattern where the only difference between species is the length of the exons. Two homeolog genes, *Xenopus laevis vgll4.L* and *vgll4.S* genes can produce two distinct mRNAs, *vgll4.La/vgll4.Lb* and *vgll4.Sa/vgll4.Sb* with a unique 5′ sequence encoded in the first two exons and a common sequence encoded in exons 3–6, the last exon containing two TDU domains (Figure S1C).

**Figure 1:**
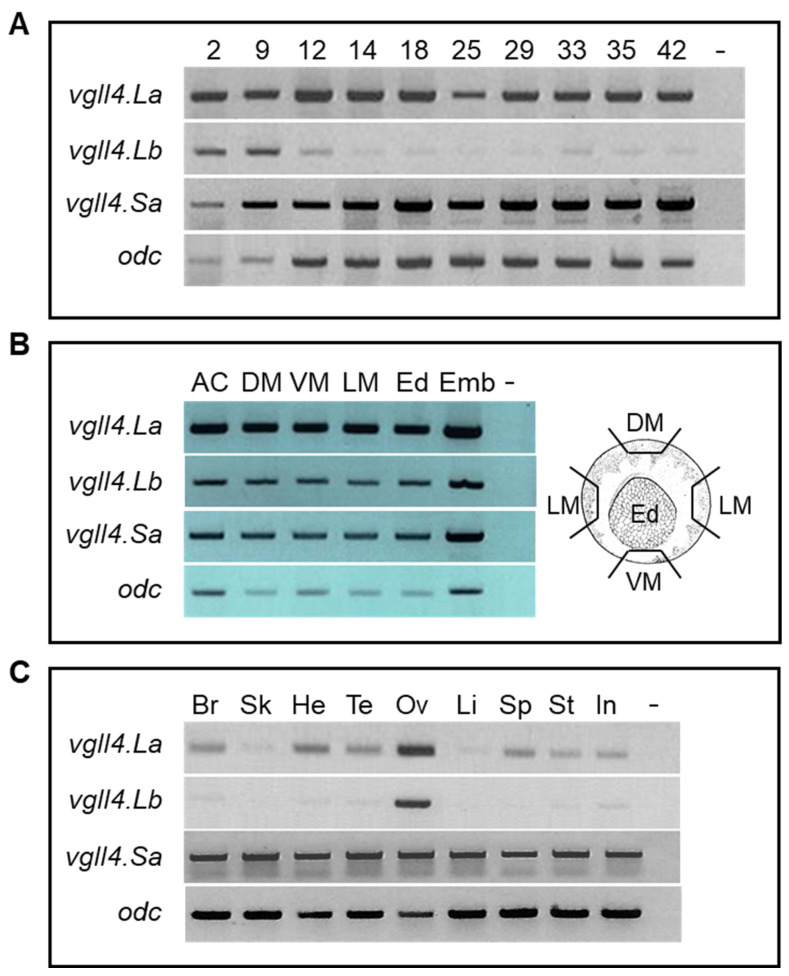
Temporal and spatial expression of *Xenopus laevis vgll4* genes. *Vgll4.La*, *vgll4.Lb* and *vgll4.Sa* mRNAs were analyzed by RT–PCR with specific primers. (**A**) RT–PCR analysis of embryos from stage 2 to stage 42. (**B**) RT–PCR analysis of dissected parts of stage 11 embryos. AC, animal cap; DM, dorsal mesoderm; Ed, endoderm; Emb, total stage 11 embryo; VM, ventral mesoderm. (**C**) RT–PCR analysis of adult tissues. Br, brain; He, heart; In, intestine; Li, liver; Sk, skeletal muscle; Ov, ovary; Sp, spleen; St, stomach; Te, testis. Ornithine decarboxylase (*odc*) gene expression was used as a control—Control without reverse transcription.

We next analyzed the expression of the *vgll4* genes via semiquantitative RT–PCR. As previously published, *vgll4.La* mRNA is maternally expressed and found at a constant level during embryogenesis (Figure 1A) [10,16]. *Vgll4.Sa* was also expressed at a constant level during development. *Vgll4.Lb* and *vgll4.Sb* mRNAs were also expressed maternally, but their levels decreased after the blastula stage and showed no detectable zygotic expression (Figure 1A). In gastrula-stage embryos, *vgll4* transcripts were found in all parts of the embryo (Figure 1B). In adult tissues, *vgll4.La* and *vgll4.Sa* were ubiquitously expressed, albeit at different levels depending on the tissues, whereas *vgll4.Lb* (and *vgll4.Sb*) was expressed predominantly in the ovary (Figure 1C). Because the two homeolog genes *vgll4.L* and *vgll4.S* have the same expression pattern and give the same morphant phenotypes (see below), *vgll4* is used hereafter for simplicity when referring to the gene.

### 3.2. Vgll4 Is Required for Early Steps of Neurogenesis

**Figure 2:**
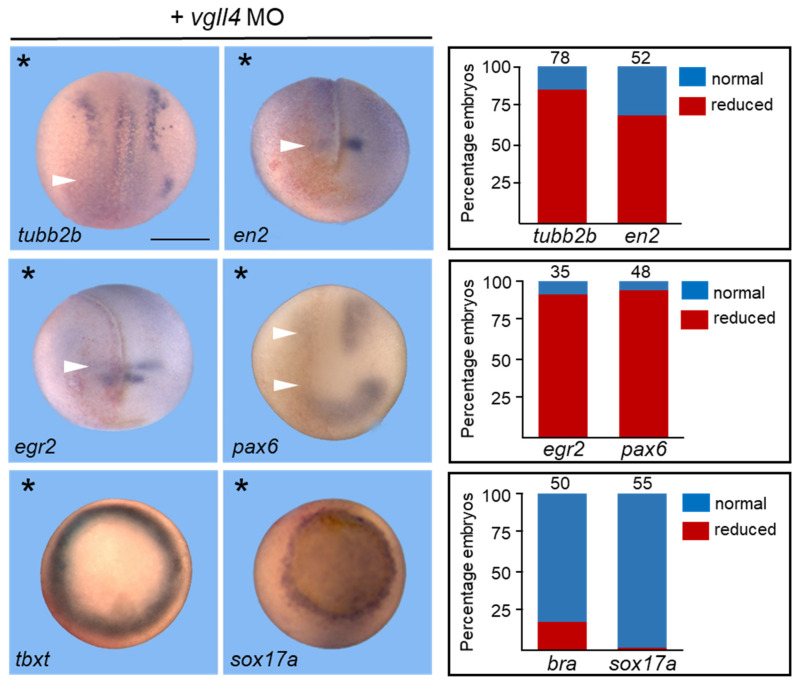
Vgll4 knockdown alters neural differentiation. Embryos were injected into one blastomere of 2-cell-stage embryos with 10 ng of *vgll4* MO and analyzed by in situ hybridization for gene expression, as indicated in the panel. mRNA encoding the lineage tracer ß-galactosidase was co-injected with the MOs to identify the injected side. The asterisk indicates the injected side. Scale bar, 500 µm. A dorsoanterior view of neurula embryos is shown. A ventral view of gastrula embryos is shown for *tbxt* and *sox17a*. Reduced gene expression is indicated by a white arrowhead. Results are shown in adjacent panels. The numbers on the top of each bar indicate the number of embryos analyzed from three independent experiments.

In neurula embryos, *vgll4* is expressed in the neural plate and neural folds [10,16]. We therefore investigated the effects of its loss of function on neural development with an antisense morpholino oligonucleotide (MO) strategy. In preliminary experiments, we found that *Vgll4.Lb* and *vgll4.Sb* mRNA knockdown had no effect on embryonic development or neuronal gene expression, as expected for genes that are not zygotically expressed. Therefore, we focused on the *vgll4.La* and *vgll4.Sa* mRNAs and designed an MO directed against each sequence (Figure S2A). Only the *vgll4 MOs*, not the control MO, reduced *vgll4* mRNA translation in the embryo (Figure S2B). Since both *vgll4.La* and *vgll4.Sa* MO provided the same results, we use vgll4 for simplicity in the remainder of the paper. In the first series of experiments, 10 ng of morpholino directed against *vgll4* mRNA were injected into one cell of 2-cell-stage embryos, and morphants were analyzed for *neural tubulin* (*tubb2b*) expression, which marks zones of primary neurogenesis. At the neurula stage, embryos injected with the control MO showed no modification of *tubb2b* expression, in contrast to vgll4-depleted embryos, in which *tubb2b* expression was dramatically reduced on the injected side, with sensitive neurons and trigeminal placode cells being affected the most (86%) (Figure 2 and Figure S3A). Sox2, an early marker of neural development, expressed in proliferative neuronal progenitors, was not affected in vgll4 morphants (Figure S3C).

We next wondered whether regional neural markers were affected by vgll4 knockdown. The expression of *en2* (midbrain-hindbrain boundary) and *egr2* (hindbrain) is severely reduced in morphant embryos (61% for *en2* and 90% for *egr2*) (Figure 2). Since *vgll4* is expressed in the optic vesicle of stage 24 embryos and later in the whole eye, we analyzed the expression of the early eye field marker *pax6*. Stage 15 embryos injected with the *vgll4* MO presented a reduction in *pax6* expression, both in the anterior and neural tube domains (94%).

All the markers tested thus far indicated that the neurectoderm layer is affected in mainly *vgll4* morphant embryos. To determine whether the effect of the vgll4 MO was germ layer specific, we analyzed morphant embryos for the expression of *brachyury* (*tbxt*) and *sox17a*, which are specific to the mesoderm and endoderm layers, respectively. *Vgll4* morphant embryos presented no sign of reduced *tbxt* (82%) or *sox17* (98%) expression (Figure 2). Neither the injection of a control MO nor a mismatch MO had any effect on the expression of any of the markers tested in any of the experiments (Figure S3A). The expression of *tubb2b* in vgll4 morphant embryos could be rescued by the injection of *vgll4* mRNA, confirming the specificity of our observations (Figure S3B). Collectively, these results support the hypothesis that vgll4 is required at an early stage of neurogenesis.

### 3.3. Vgll4 Is Required for Neurogenesis Induced by Proneural bHLH Transcription Factors

**Figure 3:**
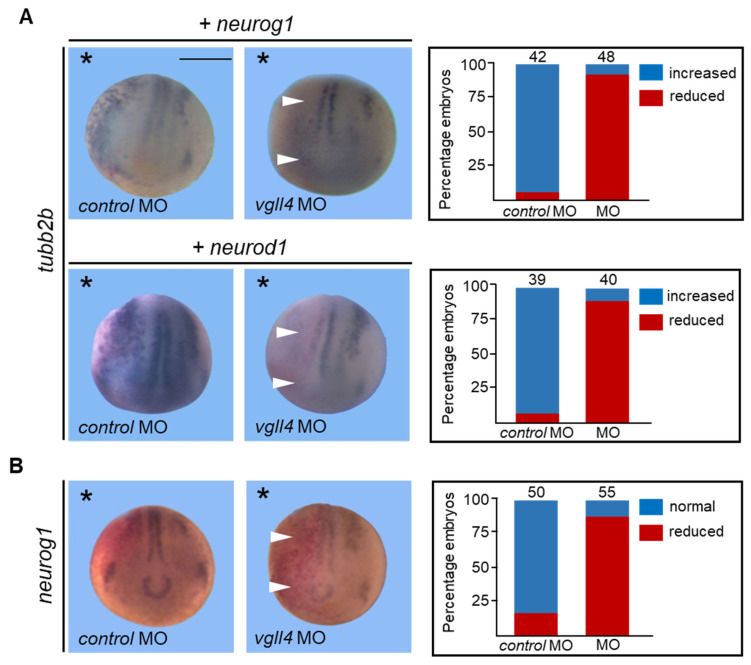
Vgll4 is required for the proneural activities of neurogenin 1 and neurod1. (**A**) Embryos were injected into one cell of a two-cell stage embryo with 1 ng of neurogenin 1 (neurog1) or neurod1 mRNA and 20 ng of control MO or with 20 ng of vgll4 MO. The embryos were analyzed via in situ hybridization for tubb2b expression at the neurula stage. mRNA encoding the lineage tracer ß-galactosidase was co-injected with the MOs to identify the injected side. (**B**) Embryos were injected into one cell at the two-cell stage embryo with 20 ng of control MO or with 20 ng of vgll4 MO and analyzed at the neurula stage via in situ hybridization for neurog1 expression. A dorsoanterior view of neurula embryos is shown. The injected side is indicated by an asterisk. Scale bar, 500 µm. Reduced gene expression is indicated by a white arrowhead. The quantification of the results is shown in adjacent panels. The numbers on the top of each bar indicate the number of embryos analyzed in three independent experiments.

Because vgll4 seems to act early within the proneural pathway, we examined whether its loss of function could affect the proneural activities of the bHLH proteins neurogenin 1 and neuronal differentiation 1 (neurod1). Embryos injected with control MO and 100 pg of *neurog1* mRNA presented an increased ectopic expression of *tubb2b* on the injected side compared with the noninjected side (Figure 3A) [43]. However, the ectopic expression of *tubb2b* was greatly reduced when 10 ng of *vgll4* MO was co-injected with *neurog1* mRNA (90%) (Figure 3A). Neurod1 acts downstream of neurogenin 1 in the neural pathway and can induce *tubb2b* ectopic expression in *Xenopus* embryos [43,44]. As expected, the injection of control MO and 200 pg of *neurod1* mRNA led to an increase in the number of *tubb2b*-positive cells on the injected side compared with the noninjected side. However, co-injection of *NeuroD1* mRNA with 10 ng of vgll4 MO blocked this increase (90%) (Figure 3A). These results suggest that the proneural activities of neurogenin 1 and neurod1 are vgll4-dependent. Since neurogenesis induced by neurogenin 1 is dependent on vgll4, we wondered whether *neurog1* expression was indeed dependent on vgll4. While the control MO displayed normal *neurog1* expression, the vgll4 MO clearly displayed a reduced *neurog1* expression (85%) (Figure 3B).

### 3.4. Vgll4 Gain-of-Function or Loss-of-Function Affects Neurogenesis

**Figure 4:**
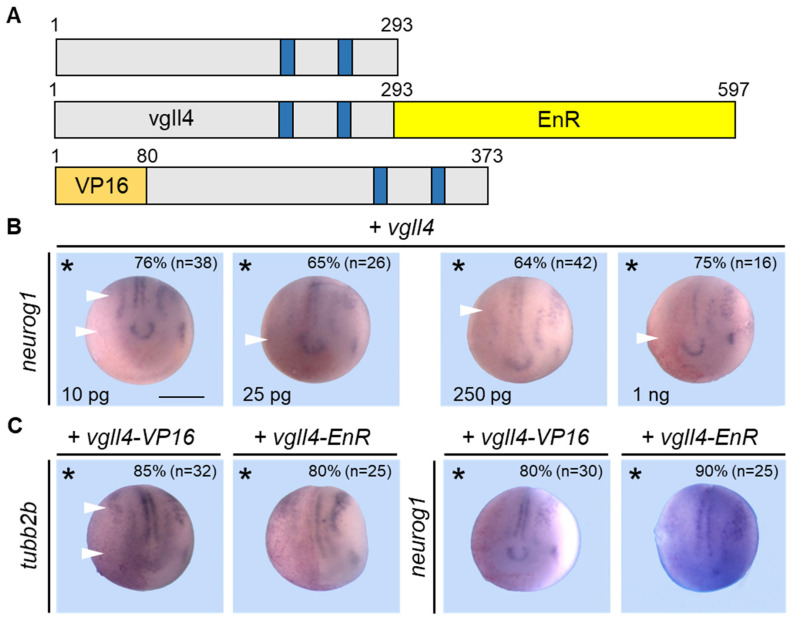
Overexpression of vgll4 blocks neurogenesis. (**A**) Schematic of the vgll4 fusion constructs. The vgll4 sequence is in gray, with TDU domains in blue. The sizes of the proteins, amino acids and different domains are indicated. EnR, repressor domain; VP16, activator domain. (**B**) Embryos were injected into one cell at the two-cell stage with increasing concentrations of *vgll4* mRNA as indicated and analyzed via in situ hybridization for *neurog1* expression. (**C**) Embryos were injected into one cell of a two-cell stage embryo with a constitutively active (VP16) or repressive form (EnR) of vgll4 and analyzed via in situ hybridization for *tubb2b* and *neurog1* expression. A dorsoanterior view of neurula embryos is shown. The injected side is indicated by an asterisk. Scale bar, 500 µm. Reduced gene expression is indicated by a white arrowhead. The quantification of the results is shown inside the panels. In (**B**,**C**), the phenotype is fully penetrant.

Vgll4 is a co-transcription factor that has no DNA-binding domain but interacts with proteins such as TEAD to regulate target gene expression. To further understand the role of vgll4 in early neural development, we undertook overexpression studies. We constructed constructs that enable the production of mRNA encoding a wild-type form of vgll4, or we fused the coding sequence of vgll4 with the Engrailed (EnR) repression domain or the VP16 activation domain (Figure 4A). Embryos were injected into one cell at the two-cell stage with increasing doses of *vgll4* mRNA and analyzed for *neurog1* expression. As shown in Figure 4B, *neurog1* expression was reduced in embryos injected with *vgll4* mRNA at a dose as low as 10 pg (76%, n = 38). Embryos injected with 25 pg (65%, n = 26), 250 pg (64%, n = 42) or 1 ng (75%, n = 16) displayed the same phenotype with reduced *neurog1* expression (Figure 4B).

We next injected either 200 pg of *vgll4-VP16* mRNA or 50 pg of *vgll4-EnR* mRNA, and analyzed neural development by *tubb2b* or *neurog1* expression. In both cases, overexpressing *V4-VP16* or *V4-EnR* mRNA reduced neuron formation on the injected side, as indicated by the repression of *tubb2b* expression (85% and 80%, respectively) (Figure 4C). The expression of *neurog1* was also reduced in embryos when either vgll4-VP16 or vgll4-EnR was overexpressed (80% and 90%, respectively) (Figure 4B). These findings indicate that vgll4 can function as a repressor or an activator to inhibit early neural pathways and neuronal differentiation.

### 3.5. Proliferation and Apoptosis Are Not Affected in Vgll4-Depleted Embryos

**Figure 5:**
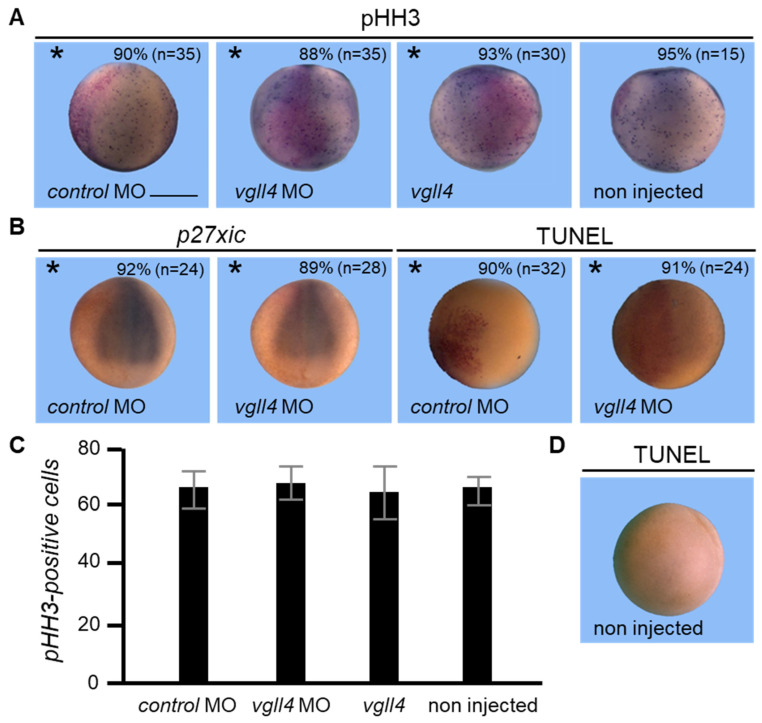
Proliferation and apoptosis are unchanged in vgll4-depleted embryos. (**A**) Embryos were injected into one cell at the two-cell stage embryo with *control* MO or *vgll4* MO and analyzed at the blastula stage by immunostaining to detect phosphohistone (pHH3). (**B**) Embryos were injected into one cell at the two-cell stage embryo with *control* MO or *vgll4* MO and analyzed at the neurula stage by in situ hybridization for *p27xic* expression and by TUNEL assay. The dorsoanterior view is shown. The injected side is indicated by an asterisk. Scale bar, 500 µm. Three independent experiments were analyzed and the quantification of the results is shown inside the panels. (**C**) Quantification of phosphohistone (pHH3). (**D**) Non-injected embryo analyzed by TUNEL staining.

Our data indicate that vgll4-depleted embryos display defects in neural development and neuronal differentiation. One possible explanation for these observations is that vgll4 could reduce the proliferation of proneural cells or, alternatively, stimulate apoptosis. To address whether the loss of *neurog1* and *tubb2b* signals is caused by reduced cell proliferation, we examined the number of cells immunoreactive for phosphohistone H3 (pHH3). Early neurula embryos injected with control MO or with 10 ng of *vgll4* MO showed no differences in pHH3 labelling between the injected side and the non-injected side (Figure 5A). Similarly, the pHH3 levels of embryos injected with *vgll4* mRNA did not differ between the injected and non-injected sides. Vgll4 morphant embryos showed no modification in the expression level of the cyclin-dependent kinase inhibitor p27^xic1^, which marks cells that are destined to become primary neurons (Figure 5B). For apoptosis analysis, embryos injected with 10 ng of *vgll4*-MO were allowed to develop to the mid-neurula stage and then assessed by TdT-mediated dUTP nick end labelling (TUNEL) staining. However, no differences in the TUNEL assay results were observed between control embryos and *vgll4* morphant embryos. Taken together, these results strongly suggest that loss of vgll4 expression reflects altered cell specification rather than proliferation/cell cycle progression defects or the death of specific cell populations.

### 3.6. Vgll4 Is Required for Neural Crest Formation and Vgll4-Depleted Embryos Develop an Abnormal Craniofacial Skeleton

**Figure 6:**
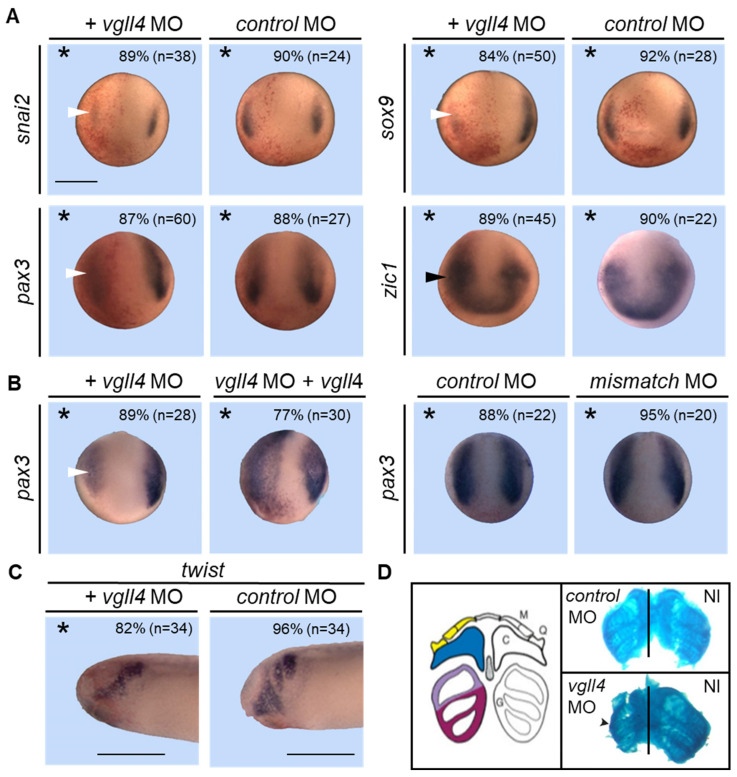
Vgll4 is required for neural crest formation and normal craniofacial development. (**A**) Embryos were injected into one cell at the two-cell stage embryo with *vgll4* MO or *control* MO and analyzed at the early neurula stage via in situ hybridization for the expression of the indicated genes. (**B**) Embryos were injected into one cell at the two-cell stage embryo with *vgll4* MO alone or with *vgll4* mRNA (*vgll4*) and analyzed at the neurula stage via in situ hybridization for *pax3* expression. Reduced gene expression is indicated by a white arrowhead and increased expression is indicated by a black arrowhead. *Control* MO or a *mismatch* MO were tested under the same conditions. A dorsoanterior view of the embryos is shown. The injected side is indicated by an asterisk. Scale bar, 500 µm. Three independent experiments were analyzed and the quantification of the results is shown inside the panels. (**C**) Embryos were injected into one cell of a two-cell-stage embryo with the *vgll4* MO or *control* MO and analyzed at the tailbud stage via in situ hybridization for *twist* expression. A lateral view of the embryo is shown. The injected side is indicated by an asterisk. Scale bar, 500 µm. The quantification of the results is shown inside the panels. (**D**) Alcian blue staining of dissected craniofacial cartilage from *vgll4* MO- or *control* MO-injected tadpoles at stage 45. The left panel is a diagram illustrating Meckel’s (M), palatoquadrate (Q), ceratohyal (C) and gill (G) cartilage. The black lines indicate the midline.

Since *vgll4* is expressed in neural crest cells, we examined whether neural crest cells were affected in vgll4-depleted embryos. We first analyzed the expression of the *snail family zinc finger 2* (*snai2*) and *SRY-box9* (*sox9*), which are two early neural crest specifiers [45,46,47]. While control MO had no effect on either *snai2* or *sox9* expression, embryos injected with *vgll4* MO presented a dramatic reduction in or a complete loss of *snai2* (89%) and *sox9* expression (84%) (Figure 6A). The neural crest arises between the neural plate and epidermis at the neural border, which is specified by a combination of transcription factors such as *pax3* and *zic1* that play key roles in pre-migratory neural crest induction [48,49,50]. *Pax3* expression is severely reduced in *vgll4* morphant embryos (87%) (Figure 6A). In contrast, the expression of *zic1*, a neural plate border specifier, was expanded in morphant embryos (89%) (Figure 6A). This suggests that the cells that did not express neural crest markers were blocked in a neural late border state and/or adopted alternative fates, such as placodes. In those experiments, the control MO or mismatch MO had no effect on the tested genes and the *pax3* morphants were rescued by co-injection of *vgll4* mRNA (77%), confirming the specificity of the results (Figure 6B).

**Figure 7:**
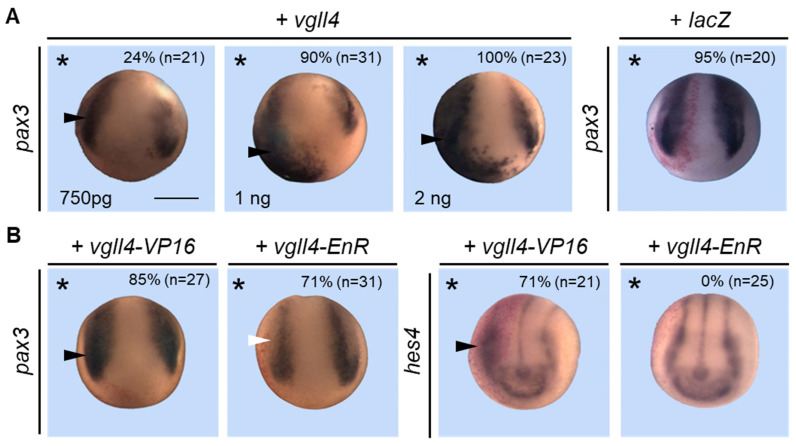
Vgll4 overexpression stimulates neural crest development. (**A**) Embryos were injected into one cell at the two-cell stage embryo with *vgll4* mRNA as indicated and analyzed at the early neurula stage via in situ hybridization for *pax3* expression. *β-gal* mRNA (*lacZ*) was injected as a control. (**B**) Embryos were injected into one cell at the two-cell stage embryo with *vgll4-VP16* or *vgll4-EnR mRNA* and analyzed at the neurula stage via in situ hybridization for *pax3* and *hes4* expression. Increased or decreased gene expression is indicated by a black or white arrowhead, respectively. A dorsoanterior view of the embryos is shown. The injected side is indicated by an asterisk. Scale bar, 500 µm. Three independent experiments were analyzed and the quantification of the results is shown inside the panels.

We next tested the effect of vgll4 overexpression on neural crest cell development. Embryos were injected with increasing amounts of *vgll4* mRNA, fixed at stage 15, stained for β-galactosidase and analyzed for *pax3* expression. The *pax3* expression domain was enlarged in embryos injected with 750 pg of *vgll4* mRNA (24%) (Figure 7A). This increase was more obvious when 1 ng of mRNA was injected (90%), which was completely penetrant with 2 ng of mRNA (100%) (Figure 7A). In control embryos, 2 ng of *lacZ* mRNA had no effect on *pax3* expression (95%) (Figure 7A). To determine whether vgll4 acts as a repressor or activator, we injected *vgll4-VP16* or *vgll4-EnR* mRNAs as previously described. Embryos injected with 200 pg of *vgll4-VP16* mRNA presented an enlargement of the *pax3* expression domain (85%), whereas embryos injected with 100 pg of *vgll4-EnR* mRNA presented a reduction in this domain (71%) (Figure 7B). Because the overexpression of either vgll4-VP16 or vgll4 expands *pax* 3 expression while vgll4-EnR reduces it, we may conclude that vgll4 functions as an activator of *pax3* expression. *Hes4* (former name *hairy*) is a downstream target of the Notch signaling target that has been shown to be required for neural crest specification [51,52,53]. We investigated *hes4* expression in embryos injected with vgll4-VP16 or vgll4-EnR mRNAs. The *hes4* expression domain is enlarged in vgll4-VP16 (71%) but is unchanged in vgll4-EnR-injected embryos (Figure 7B).

After induction, neural crest cells undergo an epithelial-to-mesenchymal transition and migrate into several locations to give rise to a wide variety of derivatives [54]. At the cranial level, the neural crest migrates through the pharyngeal arches and later contributes to cranial skeletal derivatives. Because *vgll4* is expressed in migrating neural crest cells, we further analyzed the pharyngeal arch phenotype in morphant embryos. Embryos were injected with 10 ng of *vgll4* Mo into one dorsal blastomere at the eight-cell stage and with β-galactosidase mRNA. A subset of embryos was fixed, stained and analyzed for *twist* expression, which marks the migrating neural crest. The remaining embryos were allowed to develop to stage 42 and processed for β-gal and alcian blue staining for skeletal analysis. Stage 25 morphant embryos presented reduced and altered *twist* expression on the injected side (Figure 6C). At a later stage, the morphology of morphant embryos revealed a reduction in cranial structures on the injected side compared with those on the non-injected control side, with Meckel’s and ceratohyal cartilage being affected the most (Figure 6D). Interestingly, this phenotype is very similar to that of sox9 or twist morphant embryos [45,55]. Together, these results provide strong evidence that vgll4 is required during neural crest formation in vivo and in the development of cranial neural crest derivatives.

### 3.7. Tead Morphant Embryos Phenocopy Vgll4 Morphants

**Figure 8:**
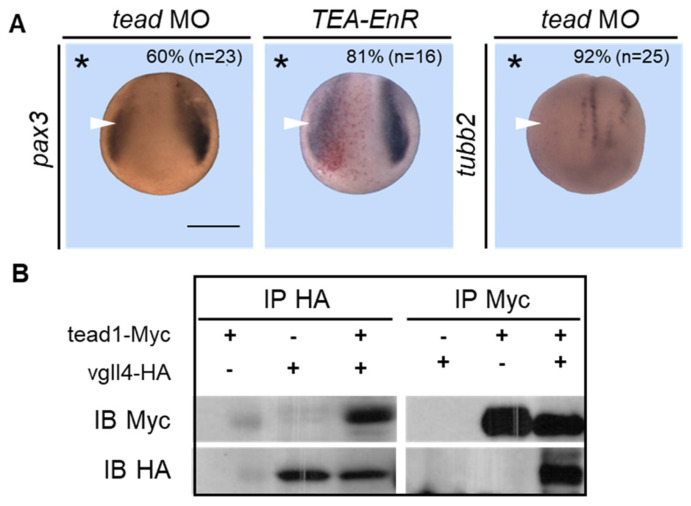
TEAD morphant embryos phenocopy vgll4 morphants. (**A**) Embryos were injected into one cell of a two-cell-stage embryo with *tead* MO or *TEA-EnR* mRNA as indicated and analyzed at the early neurula stage via in situ hybridization for *pax3* or *tubb2* expression. Reduced gene expression is indicated by a white arrowhead. A dorsoanterior view of neurula embryos is shown. The injected side is indicated by an asterisk. Scale bar, 500 µm. Three independent experiments were analyzed and the quantification of the results is shown inside the panels. (**B**) Embryos were injected into two-cell embryos at the two-cell stage with *tead1*-*Myc* alone or in combination with *vgll4*-*HA* mRNA as indicated and subjected to immunoprecipitation (IP) followed by immunoblotting (IB) with anti-Myc or anti-HA antibodies.

Vgll4 proteins are unable to bind to DNA and act as transcriptional activators or repressors through DNA-binding factors such as TEAD proteins (scalloped in *Drosophila*). There are two *tead* genes (*tead1/2*) in *Xenopus*, and we previously characterized their expression pattern, which is similar to that of *vgll4* [34]. If tead proteins are required for vgll4 functions, then embryos depleted for tead 1/2 should present the same phenotype as vgll4 morphants. To test this hypothesis, embryos were injected with 10 ng of *tead1/2* MO into one blastomere at the two-cell stage and analyzed at the early neurula stage for the expression of *pax3* and *tubb2b*. Compared with those on the control non-injected side, the number of *pax3* (60%) or *tubb2b* (92%) embryos on the injected side was lower (Figure 8A). Interestingly, embryos injected with a repressor form of tead proteins (TEA-Enr) phenocopied the injection of *Tead 1/2* MO and showed a strong reduction (81%) in *pax3* expression (Figure 8A). It has been shown that tead overexpression increases *pax3* expression in *Xenopus* neurula embryos [56]. This finding confirms that vgll4 and tead1/2 behave in the same way with respect to *pax3* expression and strongly suggests that they interact in the embryo, as expected from our current knowledge. To confirm the interaction between vgll4 and tead proteins, two blastomeres at the two-cell stage were co-injected with *vgll4-HA* and *tead1/2-Myc* mRNAs alone or in combination. At the blastula stage, the embryos were lysed, and immunoprecipitation was performed with HA or Myc antibodies. We found that vgll4 interacted with tead1 in the embryo (Figure 8B). Taken together, these results demonstrate that vgll4 can function in the embryo through its interaction with tead proteins.

### 3.8. Vgll4 Can Bypass Its Interaction with Tead for Its Activity

**Figure 9:**
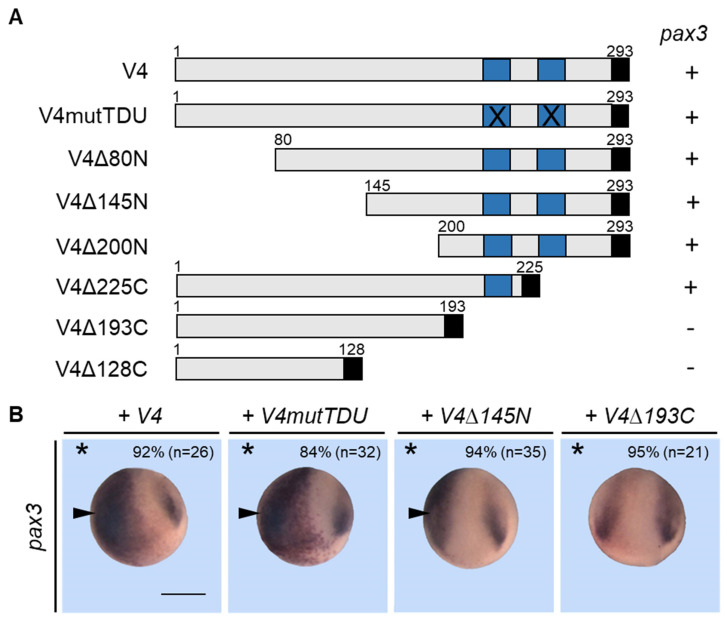
Vgll4 can bypass this interaction to activate *pax3*. (**A**) Schematic of the vgll4 (V4) fusion constructs. The sizes of the proteins with deleted regions (Δ) are indicated. The C-terminal HA tag and TDU domains are shown in black and blue boxes, respectively. In the V4mutTDU construct, both TDU domains are mutated (X). In situ hybridization of *pax3* expression in embryos injected with the different constructs is indicated on the right (+/−). (**B**) Embryos were injected into one cell at the two-cell stage embryo with the indicated vgll4 construct mRNA and analyzed at the early neurula stage via in situ hybridization for *pax3* expression. A dorsoanterior view of neurula embryos is shown. The injected side is indicated by an asterisk. Scale bar, 500 µm. Increased *pax3* expression is indicated by a black arrowhead. Three independent experiments were analyzed, and the quantification of the results is shown inside the panels.

Vgll4 proteins interact with tead proteins through their TDU domains, which have been widely characterized. The two TDU domains of vgll4 are necessary for interaction with tead, and complete inhibition of the interaction is observed when both domains are mutated in the conserved EEHF sequence [13,14]. We have shown that vgll4 can interact with tead in the embryo and that depletion of vgll4 or tead (or vgll4-EnR or TEA-EnR overexpression) induces a reduction in the *pax3* expression domain. Conversely, vgll4 or tead overexpression (or vgll4-VP16 overexpression) expanded the *pax3* expression domain. These data suggest that the protein complex vgll4-tead can activate *pax3* gene expression in the embryo. We therefore investigated whether the interaction between the two proteins is an absolute requirement for *pax3* expression. We mutated both Tondu domains in the vgll4 protein according to published works by changing the conserved EEHF sequence at the core of the domain to AAAA, abolishing the binding of vgll4 to TEAD proteins [13,14]. We also made a series of DNA constructs corresponding to either N-terminal or C-terminal deletions of the protein (Figure 9A). Embryos were injected into one blastomere of a two-cell-stage embryo with different mRNAs, collected at stage 15 and then analyzed for *pax3* expression. As expected, injection of wild-type vgll4-encoding mRNA induced strong *pax3* expression on the injected side (92%) (Figure 9B). Surprisingly, the overexpression of vgll4, which was mutated in both TDU domains, still expanded the *pax3* expression domain (84%). The overexpression of a protein deleted from its N-terminal end (V4Δ145 N) induced expansion of the *pax3* expression domain, which was equivalent to that of the wild-type protein (94%). In contrast, embryos that overexpress a deleted protein from its C-terminal end and the two TDU domains showed no modification of the *pax3* expression domain (95%) (Figure 9B). These data suggest that the ability of vgll4 to activate *pax3* is not strictly dependent on its interaction with tead but also suggests potential interactions with other transcription factors in a TDU-independent way.

### 3.9. Vgll4 Has Distinct Properties with Respect to Yap-Dependent Transcription

**Figure 10:**
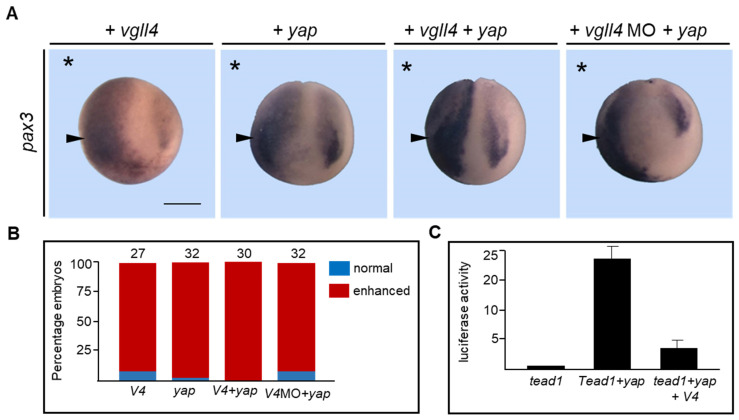
Vgll4 activity is both dependent and independent of YAP. (**A**) Embryos were injected into one cell at the two-cell stage embryo with *vgll4* (*V4*), *yap* mRNA or *vgll4* MO (V4MO) and analyzed at the early neurula stage via in situ hybridization for *pax3* expression. A dorsoanterior view of neurula embryos is shown. The injected side is indicated by an asterisk. Scale bar, 500 µm. Enhanced gene expression is indicated by a black arrowhead. (**B**) Quantification of the results. The numbers on the top of each bar indicate the number of embryos analyzed from three independent experiments. (**C**) Embryos were injected into two cells at the two-cell stage with pGL284LUC and pRL-TK DNA with the indicated mRNAs. Animal cap explants were removed at the blastula stage, and their luciferase activity was tested. Relative firefly luciferase activity (RLU) was normalized to Renilla luciferase activity. The data are presented as the means ± s.e.m.s from three independent experiments carried out in duplicate. * *p* < 0.05; Student’s *t*-test.

Tgi, the *Drosophila* homologue of vertebrate VGLL4, has been shown to compete for the binding of Yorkie (a YAP homologue) to Scalloped (a TEAD homologue), therefore inhibiting the Hippo pathway [15]. This property could confer tumor suppressor activity to VGLL4, as has been observed in several cancers [15]. We tested whether vgll4 could block yap activity in the embryo. *Yap* mRNA was injected alone or in combination with *vgll4* mRNA into one blastomere at the two-cell stage. Embryos were collected and analyzed for *pax3* expression. As expected, *Vgll4* mRNA overexpression increased *pax3* expression (90%). As previously reported, the overexpression of yap is also enhanced in the *pax3* expression domain (97%) (Figure 10A,B) [56]. When *yap* and *vgll4* mRNAs were co-injected, the enhanced *pax3* expression domain was wider and more intense than that with vgll4 or yap alone (100%) (Figure 10A,B). Because both vgll4 and yap overexpression can induce *pax3* expression, we tested whether yap could rescue vgll4 morphant embryos. *Vgll4* MO was co-injected with *yap* mRNA into one blastomere at the two-cell stage, and stage 15 embryos were subsequently analyzed for *pax3* expression. Vgll4 morphant embryos injected with yap mRNA presented *pax3* expression on the injected side, indicating that yap can rescue vgll4 depletion (Figure 10A,B).

The experiments used to demonstrate that VGLL4 competes for the YAP/TEAD interaction were performed in cell culture with a reporter gene containing TEAD binding sites [57,58]. We previously showed that the tropomyosin 1 gene is regulated in the embryo by a cis-regulatory sequence containing a TEAD binding site (or MCAT site) [41]. We used this reporter construct to determine whether vgll4 could compete for YAP/TEAD transcriptional activity. The plasmid DNA, which contains a TEAD binding site driving the luciferase gene, was injected into two blastomeres at the two-cell stage with various mRNA combinations, and the animal caps were explanted at the blastula stage before luciferase activity was measured. Embryos co-injected with *tead1* mRNA presented a very low level of luciferase activity, whereas those co-injected with both *tead1* and *yap* mRNAs presented strong luciferase activity (Figure 10C). This activity was significantly reduced when *vgll4* mRNA was co-injected with *tead1* and *yap* mRNAs (Figure 10C). Together, these experiments indicate that vgll4 can block yap-dependent transcriptional activation of a TEAD reporter gene in embryonic cells.

## 4. Discussion

In the present study, we addressed the functions of vgll4 during early development in a *Xenopus* model. We previously found that amphibian *vgll* genes are highly conserved with their mammalian counterparts [10,15]. Vgll4 has two TDU domains and is homologous to *Drosophila* Tgi, whereas the vgll1-3 proteins have a single TDU domain and are homologous to *Drosophila* Vestigial, the founding member of the Vestigial family [13,14,15]. Here, we confirm that the amphibian *vgll4* gene has the same structure as its mammalian orthologue, with two promoters and six exons. A close inspection of the databank confirmed that the avian gene also presented the same structural organization. VGLL4 expression in adult human tissues is restricted to the heart, kidney and brain, as well as other tissues, albeit at lower levels [9]. The amphibian gene is also expressed in most adult tissues, which is related to its distal promoter activity, whereas the internal promoter is active only in the ovary. During embryonic development, the *vgll4* gene is expressed at a constant level and is expressed only from the internal promoter.

### 4.1. Vgll4 Is Required for Early Steps of Neurogenesis

Since we and others have reported that vgll4 is expressed in the animal pole at the gastrula stage and then in the anterior and posterior regions of the neural plate and neural folds, we investigated its function during neurogenesis [10,16]. Vgll4-depleted embryos clearly presented an impaired neurogenesis defect, as assessed by a decrease in *tubb2* expression. In those experiments, early markers of differentiation, such as *pax6*, *en2* or *egr2*, were also downregulated. One striking observation is that the expression of the neuronal determination factor neurogenin 1 is also downregulated in vgll4 morphant embryos and that vgll4 is required for the proneural activities of both neurogenin 1 and neuronal differentiation 1 (neurod1). In those experiments, neither apoptosis nor proliferation were adversely affected, confirming that vgll4 controls the differentiation step in the process of neurogenesis. On the basis of the results of the knockdown experiments, we hypothesize that vgll4 could be an activator of both the *neurog1* and *neurod1* genes. Interestingly, we recognized several highly conserved MCAT binding sites in the 5′ upstream regions of both genes that are known to be bound by TEAD proteins (Table 1). It would be relevant to test them in transgenic assays to evaluate which of them are important for *neurog1* and *neurod1* expression. Although *vgll4* has a wide expression domain in blastula embryos, its function is clearly germ layer-specific and restricted to the neuroectoderm, as neither mesoderm nor endoderm development was impaired in our knockdown experiments.

**Figure 11:**
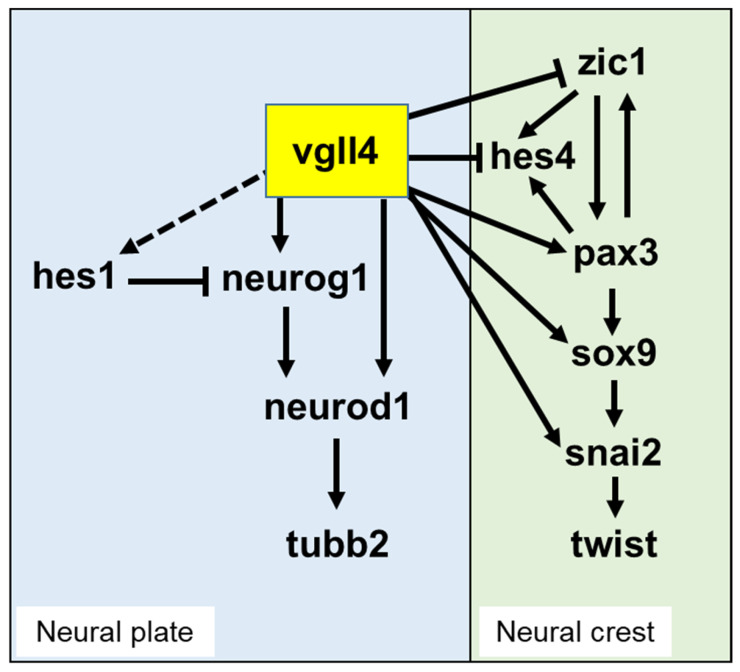
Proposed model showing the role of vgll4 in neuronal differentiation and neural crest formation in *Xenopus* embryos. The model integrates gain- and loss-of-function data from the present study and data from the literature (see text). In the neural plate, vgll4 stimulates *neurog1* expression upstream of *neurod1* and *tubb2*. Vgll4 might also directly stimulate *neurod1* expression. A possible interaction through *hes1* stimulation is shown as a dashed line. Vgll4 stimulates *pax3* expression during neural crest formation while inhibiting *zic1* and *hes4* (*hairy2*) expression. *Sox9* and *snai2* can be activated by vgll4 or as a consequence of *pax3* stimulation by vgll4.

Unexpectedly, we found that both vgll4 overexpression and knockdown impaired neurogenesis. This observation was confirmed by the overexpression of either the vgll4-VP16 or vgll4-EnR protein. One possible explanation for these results is the functional dependence on protein–protein interactions, where proper stoichiometry of each partner is essential. This is quite conceivable, as VGLL proteins are cofactors whose function relies on transcriptional partners, not only TEAD but also other proteins, such as MEF2 or ETS1, as we recently showed [11,61]. An alternative hypothesis is that vgll4 might stimulate key negative regulators of neurogenesis, such as *hes1*, a member of the HES family of transcription factors that is a downstream effector of the Notch pathway [62,63]. Interestingly, the promoter of the *Xenopus hes1* gene, as well as its mammalian orthologues, has been shown to contain a highly conserved MCAT sequence that is necessary for its enhancer functions [59]. We also identified a conserved MCAT sequence in the proximal region of the promoter (Table 1). In addition, we found that vgll4 can stimulate *hes1* expression in animal cap explants. In chickens, transfection of a TEAD-VP16 protein in the neural tube induces a reduction in neuronal differentiation concomitant with the expansion of progenitor cells [64]. This finding is compatible with our experiments in which the overexpression of the vgll-VP16 protein in *Xenopus* embryos was similar to that of TEAD-VP16 in the chicken neural system. This can explain why gain-of-function of vgll4 may block neuronal differentiation. Finally, we cannot exclude the possibility that VGLL4 interferes with the TEAD-interacting proteins that have been described thus far, some of which even play a role in the development of the nervous system [17,65]. Vgll4 might compete with these interactors for binding to TEAD, therefore regulating neuronal differentiation. Together, these data suggest that the vgll4 expression level might be critical for normal neurogenesis and that any fluctuation can have an irreversible effect on this process (Figure 11).

### 4.2. Vgll4 Is Required for Neural Crest Formation

Since we and others have previously shown that Vgll4 is expressed in neural crest cells [10,16], we addressed its potential function in the initial phase of their formation. Neural crest development relies on a complex gene regulatory network that is highly conserved between vertebrates [46,47,48]. We found that Vgll4-depleted embryos clearly presented downregulation of *snai2* and *sox9*, which are two of the earliest neural crest-specific genes expressed in pre-migratory neural crest cells [45]. These findings suggest that vgll4 acts upstream of the *snai2* and *sox9* genes. Pax3 and zic1, which are upstream of *snai2* and *sox9*, are two transcription factors whose activity is required for the formation of neural crest cells [66,67]. Vgll4-depleted embryos presented downregulation of *pax3* expression, suggesting that vgll4 regulates the earlier stage of neural crest formation. Surprisingly, the expression of *zic1* is upregulated in vgll4 morphant embryos. This might be an indirect consequence of the *pax3* downregulation observed in vgll4-depleted embryos. Indeed, the *zic1* expression domain was expanded in pax3-depleted embryos [67]. Together, our results provide a more global picture of the GRN that controls the formation of neural crest cells at the border of the neural plate in early embryos. Indeed, *vgll4* is expressed at the right time to fulfil its function in the GRN. Another possibility is that vgll4 might be considered a negative regulator of placode formation, since zic1 has been found to be a critical regulator of the formation of the pre-placodal region, the territory for the development of cranial placodes [67,68,69] (Figure 11). Once the neural crest forms, it starts to migrate to ultimately form several cranial structures in the tadpole. We found that Vgll4 morphant embryos presented a reduction in *twist*-expressing cells at the migratory stage of neural crest cells and consequently head cartilage defects as a result of *pax3*, *snai2* and *sox9* downregulation. This is reminiscent of the phenotype observed in the case of twist or sox9 depletion [45,55], confirming the role of vgll4 in the migration of neural crest cells.

Overexpression of vgll4 stimulated *pax3* ectopic expression in a dose-dependent manner. The Vgll4-VP16 protein results in the same phenotype, indicating that vgll4 functions as a transcriptional activator of *pax3*. The Vgll4-EnR fusion protein has an opposite effect to Vgll4-VP16 and downregulates *pax3* expression, confirming the effect of vgll4 on *pax3* expression through its binding to TEAD. Indeed, Gee et al. [56] identified a TEAD binding site within the 5′ regulatory region of *Xenopus pax3*, and we identified four additional sites in the 4 kb upstream sequences of *Xenopus pax3.L* gene promoter in a database search (Table 1). Moreover, Tead2 has been shown to be an endogenous activator of *Pax3* in the mouse neural crest via a minimal enhancer containing an MCAT binding site [60]. Together, these observations confirm the potential indirect role of vgll4 in *pax3* activation in neural crest cells through its binding to tead transcription factors. The role of vgll4 in neural crest cell development is also supported by the fact that *hes4* expression is stimulated in vgll4-depleted embryos. Hes4 has been found to play an important role in the specification of neural crest cells [53]. In that study, the overexpression of *pax3* or *zic1* caused the expansion of the *hes4* expression domain. This finding is in agreement with our findings that vgll4 stimulates *pax3* expression, whereas vgll4 depletion increases *zic1*, resulting in the stimulation of *hes4* expression (Figure 11). Our data align with a previously proposed GRN [70], since the expression pattern of vgll4 fits with the activation of neural crest specification via *pax 3* stimulation, whereas vgll4 has an inhibitory role on placode formation via *zic 1* inhibition (Figure 11). However, we cannot exclude a nonautonomous effect of vgll4 on the major secreted pathways Wnt, FGF and BMP [70]. Indeed, in the case of vgll3, another member of the family, we previously showed that Vgll3 can stimulate WNT and FGF expression when it is overexpressed in the embryo [61] and that it may therefore function in a non-autonomous way via those signals.

### 4.3. Vgll4 Forms a Complex with Tead in the Embryo, but This Interaction Can Be Bypassed

Vestigial-like proteins do not bind to DNA but instead form a complex with TEAD proteins to ensure their functions. We found that embryos depleted in tead1 and tead2, i.e., the two proteins expressed in the embryo, presented the same phenotype as vgll4-depleted embryos, with a reduction in *pax3* and *tubb2b* expression. Moreover, like vgll4-EnR, the fusion protein TEA-EnR reduces *pax3* expression. Furthermore, tead1 overexpression stimulates ectopic *pax3* expression, albeit faintly [56]. Together, these findings suggest that vgll4 and tead act via the same pathway. Accordingly, we confirm that these two proteins may interact in the embryo. However, this interaction was not entirely responsible for the effects observed in vgll4-depleted embryos. Mutation of the two Tondu domains in the *Drosophila* vgll4 orthologue tgi abrogates its interaction with Scalloped (the TEAD orthologue) [13,14]. Similarly, in-frame deletion of both domains prevents the interaction between human VGLL4 and TEAD [9]. In our experiments, a vgll4 protein in which the two Tondu domains were mutated still retained its ability to induce ectopic *pax3* expression. In contrast, a protein in which the N-terminal part of the protein, including the two Tondu domains, was deleted lost its *pax3*-inducing ability. We may thus conclude that vgll4 can bypass its binding to tead to activate *pax3* in the embryo, suggesting that it interacts with other yet uncharacterized transcription factors. The transcription factor MEF2 has been found to interact with human VGLL4 on one Tondu domain, whereas TEAD interacts preferentially with the second domain [9]. While we have no evidence that vgll4 can interact with mef2 in *Xenopus* embryos, this topic deserves investigation since mef2 has been found to be involved in neural crest development in mice and zebrafish [71]. Despite the demonstration by others [13,14] that VGLL4 mutated on its two TDU domains completely abolishes its binding to TEAD, we cannot exclude that these mutations in *Xenopus* embryos are not sufficient to abolish this interaction.

Another major partner protein of TEAD is the oncogene YAP, which is the effector of the hippo pathway, the complex TEAD/YAP-activating genes that stimulate proliferation and inhibit apoptosis [72,73]. In the Hippo signaling pathway, VGLL4 competes with YAP for binding to TEADs and inhibits downstream binding of YAP [24]. Similarly, we found that vgll4 can reduce the transcriptional activity of the TEAD/YAP complex in embryos via a reporter gene driven by a TEAD binding site. However, in our experiments, the overexpression of vgll4 did not reduce yap-dependent *pax3* expression. This finding is not unexpected since YAP overexpression has been shown to stimulate *pax3* expression [56]. Therefore, in our experiments, YAP overexpression could rescue *pax3* expression in vgll4-depleted embryos.

In conclusion, this is the first study to investigate the functions of vgll4 during amphibian early development. The finding that vgll4 is regulated during neural development and neural crest formation will undoubtedly lead to further investigation into its functions during mammalian development and pathologies associated with *VGLL4* expression dysregulation.

## 5. Conclusions

The present report constitutes the first attempt to decipher the functions of vestigial-like 4 (vgll4) during early vertebrate development using *Xenopus* amphibian as a model. Vgll4 is a member of a family of cofactors that can regulate gene expression by binding to TEAD transcription factors. Using gain- and loss-of-function analysis, we show that vgll4 regulates the early steps of neurogenesis and that neither neurogenin nor neurod1 are able to stimulate neurogenesis in vgll4-depleted embryos. Vgll4 also regulates neural crest development through the activation of *pax3* and *sox9* gene expression. Finally, we show that a vgll4 mutant protein, which is known to be unable to interact with TEAD, is still efficient in stimulating the *pax3* gene. Together, these findings open new avenues for analyzing the involvement of vgll4 in vertebrate development and pathologies potentially associated with its dysregulation.

## Figures and Tables

**Table 1:** TEAD binding sites. MCAT sequence corresponding to TEAD binding sites have been identified in genes potentially regulated by vgll4. The consensus sequence is in bold and its position, in base pair, relative to the translation start site is indicated.

Gene	Sequence and Position	Reference
*Neurogenin 1*	5′-**CATTCC**A-3′ (−3596/−3590)	This study
	5′-**CATTCCT**-3′ (−2298/−2292) *	
	5′-**CATTCCT**-3′ (−973/967)	
	5′-**CATTCC**A-3′ (−810/−804) *	
	5′-**CATTCCT**-3′ (−532/526)	
*Neurod1*	5′-**CATTCCT**-3′ (−1612/1606)	This study
*Hes1*	5′-**CATTCCT**-3′ (−2919/−2913) *	This study
	5′-**CATTCC**A-3′ (−50 kb) *	[59]
*Pax3*	5′-**CATTCCT**-3′ (−3923/−3917) *	This study
	5′-**CATTCCT**-3′ (−2993/−2987)	
	5′-**CATTCCT**-3′ (−2899/−2893)	
	5′-**CATTCCT**-3′ (−1649/−1643)	
	5′-A**ATTCCT**-3′ (−753/−746)	[56]
	5′-A**CATTC**A-3′	[60]
*Sox9*	5′-**CATTCCT**-3′ (−13573/−13567) *	This study
	5′-**CATTCCT**-3′ (−9267/−9261) *	
	5′-**CATTCCT**-3′ (−5872/−5866) *	
*Snai2*	5′-**CATTCCT**-3′ (−14208/−14206) *	This study
	5′-**CATTCCT**-3′ (−4558/−45526) *	
	5′-**CATTCCT**-3′ (−3329/−3323)	

* MCAT elements in reverse orientation.

## Data Availability

All data and reagents are available upon request from the corresponding author.

## Supplementary Materials

The following supporting information can be downloaded at: https://www.mdpi.com/article/10.3390/jdb14010008/s1, Figure S1: The *Vgll4* gene can produce two distinct mRNAs through alternative promoters; Figure S2: *Vgll4* translation-blocking morpholinos target *vgll4.La* and *vgll4.Sa*; Figure S3: Control morpholino effect on gene expression, and vgll4 morphant embryos.
