# Supplementary material for: Vestigial-like 4 Regulates Neurogenesis and Neural Crest Formation During Xenopus Development
Source: J Dev Biol. 2026 Feb 11;14(1):8. doi: 10.3390/jdb14010008 (PMC12922143; doi:10.3390/jdb14010008)
Supplement: Supplementary file 1 [file jdb-14-00008-s001.zip › jdb-4047763-supplementary.pdf]

Figure S1

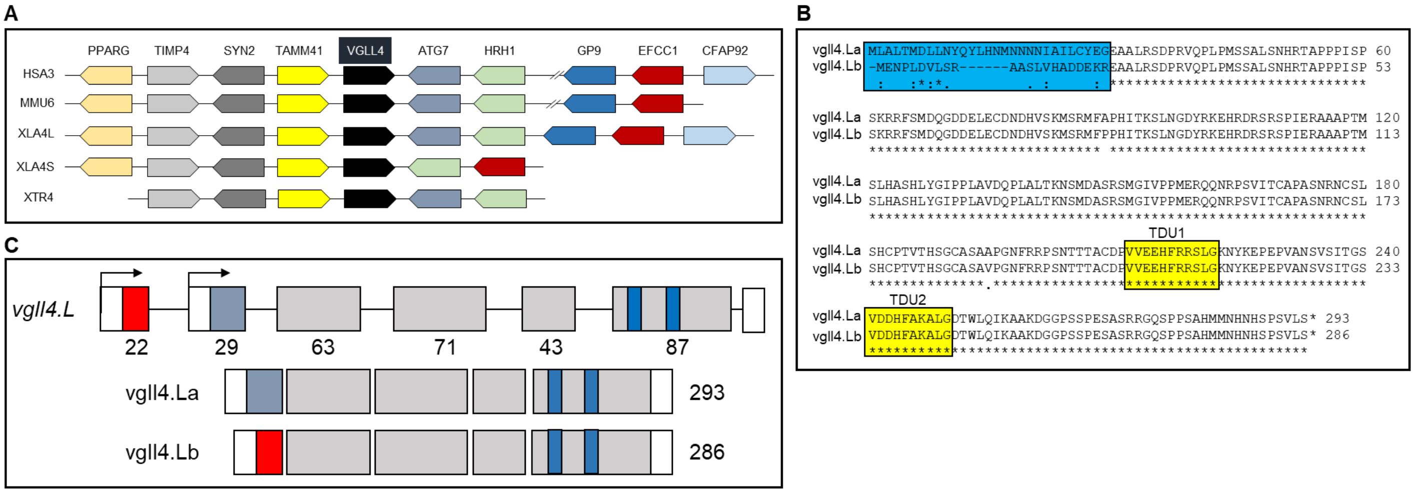

Figure S1. The *Vgll4* gene can produce two distinct mRNAs through alternative promoters. (A) Conserved syntenic regions between the human (HSA), mouse (MMU), *Xenopus laevis* (XLA) and *Xenopus tropicalis* (XTR) chromosome regions containing the *VGLL4* locus. Gene symbols are according to HUGO. The chromosome number for each species is indicated, as are the two subgenomes of *Xenopus laevis* (XLAL and XLAS). Genes are represented as colored boxes, with the arrow indicating the orientation of the transcription unit. Boxes with the same color correspond to orthologous genes. The following versions of the genomes used for establishment are as follows: human, GRCH38; p7; mouse, GRCm38; and *Xenopus laevis* version 9.1. The drawing is not on scale to avoid complexity. (B) Deduced amino acid sequence comparison between the vgll4.La and vgll4.Lb proteins. Distinct N-terminal sequences are boxed in blue and TDU domains are boxed in yellow. Identical and conserved amino acids are indicated by an asterisk or a dot, respectively. (C) Structure of the *Xenopus laevis vgll4.L* gene and its corresponding mRNAs. Common exons are in grey boxes, the untranslated region is in white, and the intron is represented by a black line. The amino acid-deduced exon size is indicated under the exons.

Figure S2

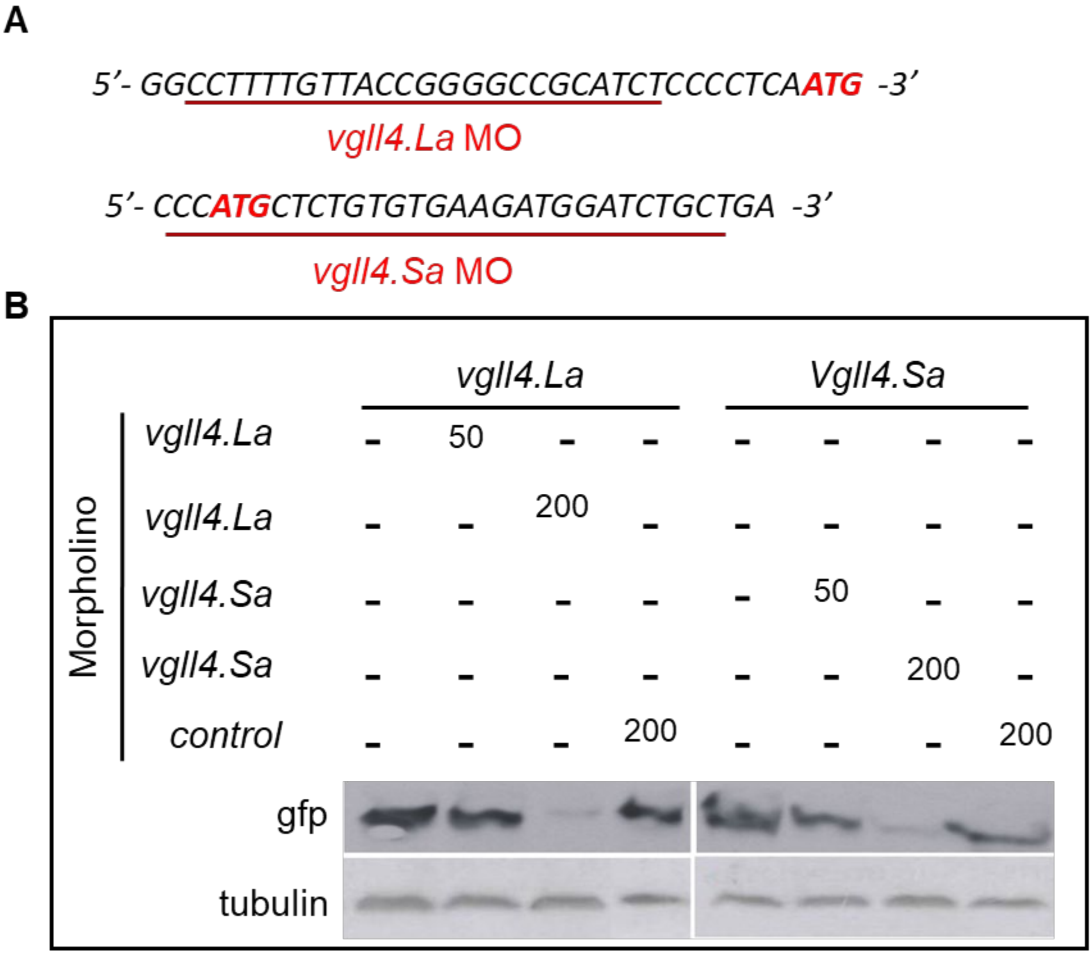

Figure S2. (A) *Vgll4* translation-blocking morpholinos target *vgll4.La* and *vgll4.Sa*. The position of the MO with respect to the 5' sequence of the mRNA is indicated by a red line. (B) Western blot analysis with anti-GFP using lysates from embryos injected with 50 or 200 pg of *vgll4.La* or *vgll4.Sa. Sa* mRNA alone or in combination with the indicated morpholino. Tubulin is shown as a loading control.

Figure S3

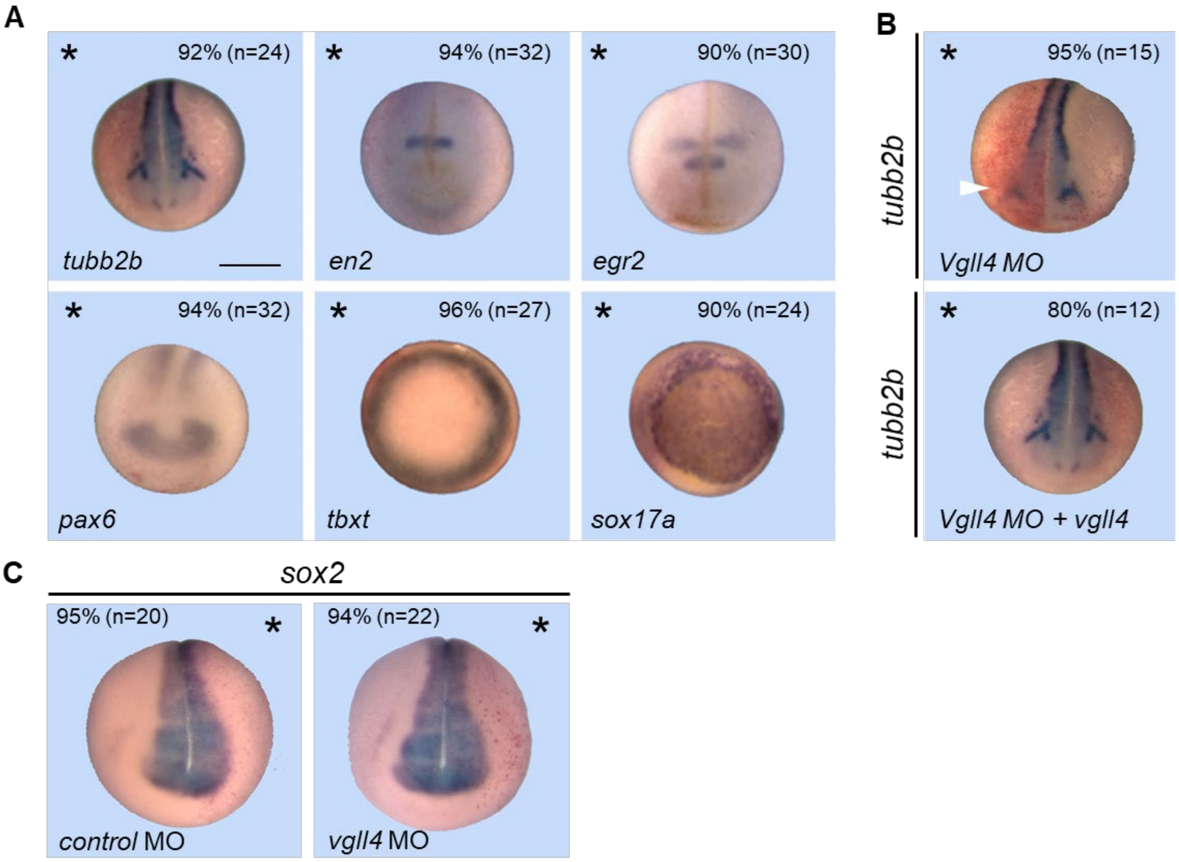

Figure S3. Control morpholino effect on gene expression, and vgll4 morphant embryos. (A) Embryos were injected into one cell at the two-cell stage embryo with *control* MO and analysed by *in situ* hybridization for the expression of the indicated genes. A dorsoanterior view of the embryos is shown for *tubb2, en2, egr2* and *pax6,* and a ventral view is shown for *tbxt* and *sox17a*. (B) Embryos were injected into one cell of a two-cell-stage embryo with *vgll4* MO in the absence or presence of *vgll4* mRNA and analyzed *via in situ* hybridization for *tubb2* gene expression. The injected side is indicated by an asterisk. Reduced gene expression is indicated by a white arrowhead. Scale bar, 500 µm. Three independent experiments were analysed, and the quantification of the results is shown in the panels. (C) Embryos were injected into one cell of a two-cell-stage embryo with the *control* MO, or *vgll4* MO, and analyzed by *in situ* hybridization for *sox2* gene expression.
